# THIS IS THE YEAR THAT WAS: THE BEST OF THE JOURNAL OF GERONTOLOGY BIOLOGICAL SCIENCES

**DOI:** 10.1093/geroni/igae098.0563

**Published:** 2024-12-31

**Authors:** Gustavo Duque, Laura Haynes

**Affiliations:** Chair; Co-Chair

## Abstract

This session features the authors of the most influential papers published in the Journal of Gerontology, Biological Sciences, based on the highest impact in 2023. The authors will present the most exciting findings of the year on aging biology. Six authors of Editor’s Choice papers will be invited to summarize their paper and the future implications of their discoveries for 10 minutes, followed by 5 minutes of Q&A from the audience.

